# Motor Responses of Lumbar Erector Spinae Induced by Electrical Vestibular Stimulation in Seated Participants

**DOI:** 10.3389/fnhum.2021.690433

**Published:** 2021-07-22

**Authors:** Amélie Desgagnés, Mikaël Desmons, Jean-Philippe Cyr, Martin Simoneau, Hugo Massé-Alarie

**Affiliations:** ^1^Centre Interdisciplinaire de Recherche en Réadaptation et en Intégration Sociale (CIRRIS), Laval University, Quebec City, QC, Canada; ^2^Kinesiology Department, Laval University, Quebec City, QC, Canada; ^3^Rehabilitation Department, Laval University, Quebec City, QC, Canada

**Keywords:** electrical vestibular stimulation, back muscles, erector spinae, vision, postural control

## Abstract

**Introduction:** The study of motor responses induced by electrical vestibular stimulation (EVS) may help clarify the role of the vestibular system in postural control. Although back muscles have an important role in postural control, their EVS-induced motor responses were rarely studied. Moreover, the effects of EVS parameters, head position, and vision on EVS-induced back muscles responses remain little explored.

**Objectives:** To explore the effects of EVS parameters, head position, and vision on lumbar erector spinae muscles EVS-induced responses.

**Design:** Exploratory, cross-sectional study.

**Materials and Methods:** Ten healthy participants were recruited. Three head positions (right, left and no head rotation), 4 intensities (2, 3, 4, 5 mA), and 4 EVS durations (5, 20, 100, 200 ms) were tested in sitting position with eyes open or closed. EVS usually induced a body sway toward the anode (placed on the right mastoid). EMG activity of the right lumbar erector spinae was recorded. Variables of interest were amplitude, occurrence, and latency of the EVS-induced modulation of the EMG activity.

**Results:** The short-latency response was inhibitory and the medium-latency response was excitatory. Increased EVS current intensity augmented the occurrence and the amplitude of the short- and medium-latency responses (more inhibition and more excitation, respectively). EVS duration influenced the medium-latency response differently depending on the position of the head. Right head rotation produced larger responses amplitude and occurrence than left head rotation. Opposite head rotation (left vs. right) did not induce a reversal of the short- and medium-latency responses (i.e., the inhibition did not become an excitation), as typically reported in lower legs muscles. The eyes open condition did not modulate muscle responses.

**Conclusion:** Modulation of EVS parameters (current intensity and duration of EVS) affects the amplitude and occurrence of the lumbar erector spinae responses. In contrast, vision did not influence the responses, suggesting its minimal contribution to vestibulomotor control in sitting. The lack of response reversal in sagittal plane may reflect the biomechanical role of lumbar erector spinae to fine-tune the lumbar lordosis during the induced body sway. This hypothesis remains to be further tested.

## Introduction

The vestibular system contributes to balance control and posture (Gandevia et al., 2012; Forbes et al., 2014; Kingma and van de Berg, 2016). The central integration of sensory inputs, such as vestibular, proprioceptive and visual, ensures adequate motor control to estimate self-motion and maintain a stable posture (Cullen, 2011; Gandevia et al., 2012). Electrical vestibular stimulation (EVS) (previously known as galvanic vestibular stimulation) (Dlugaiczyk et al., 2019; Sluydts et al., 2020) is a technique used to study the role of the vestibular system in postural control (Dlugaiczyk et al., 2019; Ertl and Boegle, 2019). The application of transcutaneous current changes the polarization of the eighth cranial nerve, resulting in the modulation of the activity of the vestibular afferents without the need to move the head (Fitzpatrick and Day, 2004; Ertl and Boegle, 2019). EVS modulates the continuous firing rate of vestibular afferent fibers and probably recruits vestibular hair cells (Gensberger et al., 2016): the firing rate increases on the cathodal side (depolarization) and decreases on the anodal side (hyperpolarization) (Fitzpatrick and Day, 2004). When applied in a standing position, EVS creates an illusion of movement toward the cathode and a postural sway toward the anode (Lund and Broberg, 1983; Fitzpatrick and Day, 2004) along the interaural line (Fitzpatrick and Day, 2004). The postural response may result from a counteraction from the balance system to maintain balance; it would be opposite to the EVS-perceived body sway (Fitzpatrick et al., 1994; Fitzpatrick and Day, 2004). Thus, EVS allows studying the postural response and its related electromyographic (EMG) activity (Fitzpatrick and Day, 2004). EVS induces motor responses when muscles are engaged in balance control (e.g., the *soleus* in standing) (Britton et al., 1993; Ali et al., 2003; Dakin et al., 2016). The typical EVS-induced motor responses have two components: a short-followed by a reversed medium-latency response (i.e., inhibitory and excitatory) (Britton et al., 1993). The direction of the EVS-induced response (e.g., inhibition or excitation) depends on the position of the anode and head orientation in relation to the body. For example, EVS applied while standing with the head rotated to the right and the anode positioned on the right mastoid process produces a backward body sway. In both *soleus* muscles, the resulting response corresponds to a short-latency inhibition followed by a medium-latency excitation in EMG activity (Britton et al., 1993). In contrast, rotating the head to the left while keeping the anode on the right mastoid process produces a forward body sway and reverses the direction of the responses (i.e., the short-latency becomes excitatory, and the medium-latency, inhibitory). Finally, when EVS is used while participants look forward, a body sway occurs toward the anode (i.e., in the frontal plane). Thus, EVS induces a sway in a particular direction (e.g. backward, forward, right, or left) depending on the head position.

Although back muscles are crucial in the control of balance and posture (Massion, 1992), few studies tested the effect of EVS on back muscles activation. There is emerging evidence that differences exist between limb and trunk vestibular control, which implies that the optimal stimulation parameters may differ when studying trunk muscles compared to lower limb muscles. First, a recent study highlighted that the EVS-induced responses in thoracic and lumbar *erector spinae* (LES) muscles were less flexible than those of *soleus* muscle (Guillaud et al., 2020). Although tasks reducing the contribution of the *soleus* in balance control (e.g., standing with head contact on a vertical panel compared to without) also attenuated the *soleus* responses amplitude, such modulation did not occur for LES motor responses. Also, Guillaud et al. (2020) did not observe the reversal of these responses in back muscles (i.e., short-latency, inhibitory vs. medium-latency, excitatory) when comparing EVS-induced body sway in anterior and posterior directions, although another study reported reversal in the frontal plane for at least one participant (Ali et al., 2003). Second, the phase frequency estimates of LES motor responses was smaller than in lower limb and neck muscles responses during a stochastic vestibular stimulation (i.e., an unpredictable current used to study the vestibular system) (Dakin et al., 2007; Forbes et al., 2013). The authors suggested that this could represent a limited functional contribution of the LES muscles compared to lower limb and neck muscles for controlling balance in standing (Forbes et al., 2014). Third, it was suggested that the amplitude of EVS-induced motor responses in back muscles was considerably smaller than in limb muscles (Ali et al., 2003). These results highlight the importance of studying the influence of parameters on EVS-induced responses especially in back muscles in relevant postural conditions (i.e., when back muscles are more likely to be involved).

Although three studies tested the LES motor responses during EVS (Ardic et al., 2000; Ali et al., 2003; Guillaud et al., 2020), there is still an important knowledge gap about the influence of EVS parameters on these motor responses. Only one study has tested the effect of EVS intensities (up to 4 mA). The authors reported that using EVS intensity of more than 2–2.5 mA induced short-latency response of greater amplitude than smaller intensity although it was not clarified if this was for the *soleus* or LES muscles, and no descriptive statistics were reported (Ali et al., 2003). Other EVS studies of LES muscles used only one stimulation intensity [0.6 mA (Ardic et al., 2000) or 3.5 mA (Guillaud et al., 2020)]. EVS intensity between 1 mA (Britton et al., 1993) or 6 mA (Fitzpatrick et al., 1994) were used in studies of neck and limbs muscles although the rationale was not justified. Also, although very-short duration of EVS (e.g., 2 ms) evoked short-latency response in sternocleidomastoid (Watson and Colebatch, 1998) and masseter (Deriu et al., 2003) muscles, most EVS studies of back muscles used relatively longer EVS duration [e.g., 175 ms (Guillaud et al., 2020), 400 ms (Ali et al., 2003)]. The use of a long duration stimulus complicates the interpretation of the measured latency since it is not possible to determine when nerve depolarization occurs. Thus, it is unclear what are the optimal parameters to evoke motor responses in LES muscles.

The balance task tested may modulate the EVS-induced response. For example, a less demanding task in terms of postural control for ankle muscles attenuates the response amplitude (Fitzpatrick et al., 1994; Guillaud et al., 2020). Besides, when balance control involves the upper limb (e.g., holding a handle), responses appear in the *triceps brachii* muscles (Britton et al., 1993). These examples suggest high task-dependent flexibility of the vestibular system to maintain balance. Testing EVS-induced responses in trunk muscles in a sitting position appears relevant considering their important contribution to balancing the upper body and the possibility to by-pass the contribution of lower leg muscles to maintain a stable posture in this position. Although one study reported responses in LES muscles in a sitting position and reported a smaller response than in standing, many methodological factors such as the lack of EMG activity control during postural tasks makes this result difficult to interpret (Ali et al., 2003). In addition, using a sitting position may enable to combine EVS with other neurophysiological techniques such as transcranial magnetic stimulation, in which participants are seated when testing back muscles (O’Connell et al., 2007; Tsao et al., 2011a,b; Massé-Alarie et al., 2013, 2016a,b; Masse-Alarie et al., 2018). Combining these techniques may help to better understand the central processing of the vestibular responses.

Visual cues contribute to upright and seated postural control (Day and Cole, 2002; Blouin et al., 2007). For example, occluded vision increases body sway and related EVS-induced responses (Fitzpatrick et al., 1994; Mackenzie and Reynolds, 2018). However, it is still unknown if the absence/presence of vision similarly influence LES motor responses in sitting. The availability of more tactile inputs from the thighs in sitting compared to standing could differently impact motor responses induced by EVS.

Overall, it remains unclear how the modification of EVS parameters (e.g., EVS current intensity and EVS duration), head rotation or vision influence the short- and medium-latency LES motor responses. This study aimed to explore the effect of EVS parameters and conditions on LES motor responses in sitting. The main objective was to determine the effect of head position, current intensity and duration of EVS current on the amplitude, latency and occurrence of the short- and medium-latency LES motor responses. A secondary objective was to determine the effect of vision on the amplitude, latency and occurrence of the short- and medium-latency LES motor responses. Considering that a condition (i.e., a combination of parameters) usually producing a backward sway was associated with larger activation of LES muscles, we hypothesized that the head position (usually producing a backward sway) would produce larger amplitude and more frequent occurrence of LES motor responses as well as a higher EVS current intensity, a longer EVS duration and an absence of vision.

## Materials and Methods

### Participants

The study involved a convenience sample of ten healthy participants recruited from the *Centre Interdisciplinaire de Recherche en Réadaptation et en Intégration Sociale* (CIRRIS) in Quebec City from July 9th, 2019 to August 28th, 2019. To be eligible for the study, participants needed to be between 18 and 60 years old. Exclusion criteria were: (i) pathology of the vestibular system (e.g., Menière’s disease, benign paroxysmal positional vertigo), (ii) pregnancy, (iii) allergy to tetracaine (the protocol involved the use of tetracaine-based analgesic cream), (iv) back pain, (v) idiopathic scoliosis, and (vi) any major pathologies interfering with the task tested in this study (e.g., neuropathy). We chose to exclude participants with scoliosis because of the imbalance in response to EVS between the right and left vestibular systems observed in these participants (Pialasse et al., 2015, 2016; Hatzilazaridis et al., 2019). The study was approved by the Ethics Research Committee of the *Centre Intégré Universitaire de Santé et de Services Sociaux de la Capitale-Nationale* (Project: #2019-1778), in accordance with the Declaration of Helsinki. All participants provided their written informed consent prior to the experiment.

### Experimental Design

To test the effect of head position, EVS current intensity and EVS duration on the LES motor responses (main objective), participants sat on a chair without backrest with their arms along the body, feet touching the floor, and eyes closed. Participants had to maintain 15 ± 5% of the maximal voluntary contraction (MVC) of the right LES muscles by performing a slight active anterior pelvic tilt. Since participants had their eyes closed in most conditions, online auditory feedback helped maintaining a steady contraction using the pre-amplified EMG signal translated into an audio signal played through speakers. Online visual feedback [i.e., root mean square (rms) EMG] also helped the investigators to ensure muscle contractions accuracy. The investigators provided verbal feedback when necessary (i.e., instructions to increase or reduce the contraction). A period of training allowed participants to familiarize themselves with the procedure. Muscle activation was used to (i) standardize the pre-stimulus background EMG between participants and (ii) increase motoneuron excitability during EVS (Di Lazzaro et al., 1998). EVS studies consistently reported responses in muscles actively engaged in maintaining postural control [e.g., no responses in *tibialis anterior* muscle at rest (Fitzpatrick et al., 1994) or in *soleus* muscle in sitting participants (Ali et al., 2003)].

**FIGURE 1:**
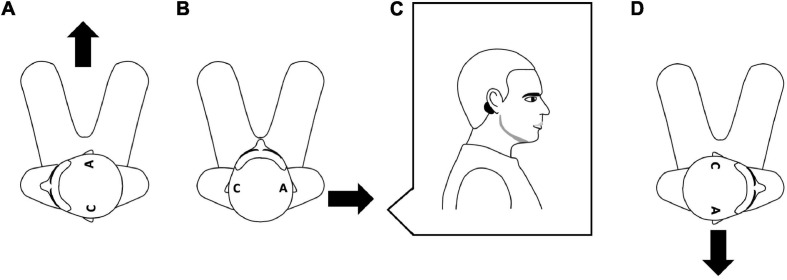
Relationship between head rotation, electrodes position and usual direction of the induced body sway. The figure presents an aerial view of the seated participant. The letter “C” indicates the position of the cathode and the letter “A” indicates the position of the anode, and the black arrows indicate the direction of the induced body sway. Left head rotation with the anode on the right mastoid induces a forward sway **(A)**, no head rotation with the anode on the right mastoid usually induces a right body sway **(B)** and right head rotation with the anode of the right mastoid induces a backward sway **(D)**. **(C)** Depicts a side view of the participant to show the position of an electrode (black circle) on the mastoid process.

Participants came to the laboratory for one session of ∼3 h. For the main objective, 48 conditions were tested for this part of the study. Each condition combined parameters of EVS current intensity (2, 3, 4, and 5 mA), EVS duration (5, 20, 100, and 200 ms), and head rotation (maximal comfortable right and left rotations, and no rotation). Figure 1 presents the relationship between head rotation, electrodes positioning and usual induced body sway. For each condition, participants had their eyes closed and received a sequence of 15 stimulations, for a total of 720 stimulations. Fifteen stimulations per condition was chosen after the realization of pilot experiments to have an optimal balance between good signal-to-noise ratio and the duration of the session. EVS was randomly triggered between 5 and 8 s with respect to the last stimulation. The order of the conditions was randomized using two steps: (i) in function of the rotation of the head then (ii) in function of current intensity. The order of the EVS duration was not randomized and was always tested from the shortest to the longest EVS duration (5–200 ms). The condition with eyes open was tested after the same condition with eyes closed. Chin-acromion distance was measured to standardize the head rotation amplitude within-participant. In this way, the ranges of motion of right and left rotations were equal for a same participant. Participants maintained the same position throughout the experiment and avoided touching their legs or trunk since a light touch reduces the amplitude of LES motor responses (Maaswinkel et al., 2014). Rest periods were given as needed.

To test our secondary objective on the effect of vision on LES responses (secondary objective), participants completed two additional conditions with eyes open: (i) right head rotation and (ii) left head rotation (EVS current intensity: 4 mA, EVS duration: 200 ms). Due to time constraint and feasibility, we did not repeat all conditions with eyes open and selected parameters based on published EVS studies and on our results from pilot experiments. The position was the same as described previously.

Overall, participants received a total of 750 stimulations in 50 different conditions. The only adverse effect reported was a slight fatiguability of back muscles at the end of the session for some participants.

### EMG and Maximal Voluntary Contraction

A pair of Ag/AgCl surface electrodes (Kendall Medi-trace 200, Covidien, Dublin, Ireland) was placed 2 cm lateral to the L3-L4 joint line onto the right LES belly following SENIAM recommendations (Hermens et al., 2000). A ground electrode was positioned on the right anterior superior iliac spine and iliac crest. EMG of leg muscles was not recorded considering that no response was observed in sitting (Ali et al., 2003; Fitzpatrick et al., 1994; Day et al., 1997). In sitting position, participants performed a MVC during 3 s. in anterior pelvic tilt and in resisted trunk extension at the thoracolumbar spine junction. MVC of the task producing most EMG activity in the right LES muscle was tested again twice to measure three MVCs using the same technique. This method was used since (i) some individuals are not able to produce MVC in anterior pelvic tilt or in resisted trunk extension (e.g., flexion of the lumbar spine during resisted trunk extension or inability to perform the anterior pelvic tilt) and (ii) it reduced the likelihood to underestimate the MVC. A smaller MVC will result in a smaller absolute value for the 15% MVC during the task and will potentially result in smaller responses to EVS. The evaluator provided instructions and demonstrations on how to perform these contractions. To ensure maximal contraction, the evaluator motivated each participant. Thirty seconds resting periods separated MVC trials. The largest peak of rms EMG was considered as MVC, regardless of the technique (i. e., pelvic tilt vs. resisted lumbar extension). EMG was bandpass filtered (10–500 Hz, bandwidth filter), amplified 1,000 times (NEO-210A Analog Output Module, NTI) and sampled at 1,000 Hz using Power 1401 Data Acquisition System and Spike2 software (Cambridge Electronic Design, Cambridge, United Kingdom).

### Electrical Vestibular Stimulation

Application of tetracaine hydrochloride gel, i.e., anesthetic cream (AMETOP GEL 4%, Smith and Nephew Medical Ltd., Hull, United Kingdom) on both mastoid processes 30 min before installing the electrodes reduced nociceptive and tactile sensations produced by EVS (Ertl and Boegle, 2019). The duration of the 750 stimulations, regardless of the preparation period, the time to adjust the parameters and the pauses took approximately 80–90 min. A bipolar constant current stimulator (Digitimer DS5, United Kingdom) produced a binaural EVS (using square-wave pulse current, i.e., the intensity is the same throughout the stimulation duration) through round electrodes (3.2 cm diameter PALS, Axelgaard Manufacturing Co., Fallbrook, United States) placed on the right (anode) and left (cathode) mastoid processes. Participants wore a headband for optimal contact between the electrodes and skin. Square wave pulses were generated with the Spike2 software using the Graphical Editor Tool, that enables modifying current intensity, duration, and waveform, and to trigger externally the electrical stimulation (i.e., the DS5).

### Data Extraction and Analysis

For each short- and medium-latency response, three variables were calculated: latency, rms EMG amplitude and occurrence in the software Spike2. For each condition, the average rectified EMG was analyzed. The averaged EMG comprised the signal of the 15 stimulations gated to stimulus events. Via a microcontroller (model Leonardo, Arduino) the output of the constant current stimulator produced a TTL pulse serving as an input to the data acquisition board. The event served as a reference (time zero) for the response latency. Studied time windows ranged from −200 to 300 ms around the event onset. A two-step process was used to quantify the amplitude of the motor responses depending on if a response was discernible or not. First, when a motor response was discernible, the onset and offset of the short- and medium-latency motor responses was determined visually. EVS, however, did not always evoke a discernible LES motor response, and in the first step of this analysis, the evaluator skipped these conditions. When all the conditions were analyzed, the average onset and offset of the discernible motor responses were used to determine time windows for both short- and medium-latency motor responses. Second, these time windows were used to measure rms amplitude in the conditions without discernible responses i.e., 45–70 and 75–100 ms for short- and medium-latency responses, respectively. This technique provided quantitative values in the absence of discernible motor responses. The amplitude of the motor response was calculated as a percentage of the MVC after subtracting the pre-stimulus rms EMG (from –110 to –10 ms). This method allowed determining the direction of the response (excitatory vs. inhibitory) relative to the participant’s maximal contraction (Massé-Alarie et al., 2019). The occurrence was calculated as the percentage of participants having a discernible inhibitory/excitatory motor response (on the average rectified EMG signal of the 15 stimuli) on the total number of participants tested. The occurrence was measured for each condition. For example, a condition eliciting a discernible motor response in 8 out of 10 participants had an occurrence of 80%. The presence/absence of a motor response was determined visually. Published studies used similar visual strategies to identify motor-evoked potentials of back muscles using transcranial magnetic stimulation (Massé-Alarie et al., 2013; Masse-Alarie et al., 2016, 2018), withdrawal nociceptive reflex using electrical noxious stimulus of trunk muscles (Masse-Alarie et al., 2019) and anticipatory postural adjustment (Hodges and Bui, 1996).

### Statistical Analysis

The statistical analysis was performed on SPSS (version 26 Premium, IBM corp., United States). Shapiro-Wilk’s tests assessed the normality of the distribution. However, despite transformations, it was not possible to achieve a normal distribution. Thus, linear mixed models were realized on non-transformed data. Significance was set at *p* < 0.05. Results are presented as [mean (SD)].

The statistical model tested the rms EMG amplitude (% MVC) and the occurrence of the responses for both objectives. Due to many missing values for the latency (i.e., no discernible motor response), latency was described quantitatively without statistical analysis. To determine the effect of head position, EVS current intensity and EVS duration on the amplitude and occurrence of the short- and medium-latency motor responses (main objective), linear mixed models were independently computed on the short- and medium-latency variables. The model used a *scaled identity* covariance matrix with Rotation (right, left, no), EVS duration (5, 20, 100, 200 ms), and Current intensity (2, 3, 4, 5 mA) as fixed factors, and participants’ intercept as the random factor (i.e., to consider the variability between participants’ measurements). To determine the effect of vision on the responses of LES muscles (secondary objective), linear mixed models were independently computed on the short- and medium-latency variables. The model used a *scaled identity* covariance matrix with Rotation (right or left) and Vision (eyes open or closed) as fixed factors and participants’ intercept as the random factor. Sidak’s test corrected for multiple pairwise comparisons.

## Results

**FIGURE 2:**
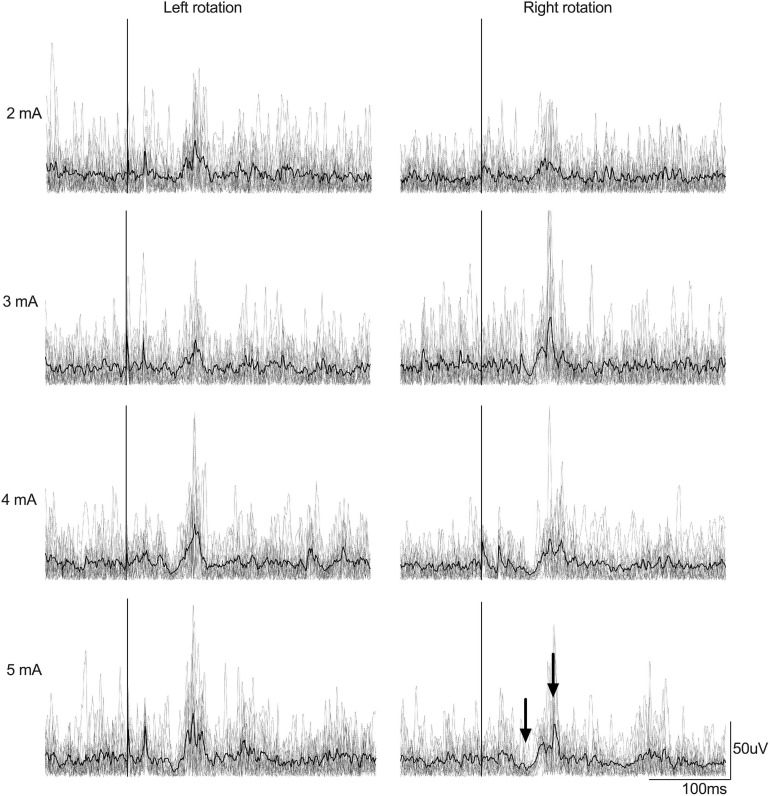
Example of rectified EMG signals recorded from the right LES muscles from a participant according to EVS intensity (EVS duration was 20 ms). Left column depicts muscle amplitude for left head rotation and right column show muscle amplitude for right head rotation. Gray lines represent the EMG traces recorded for each 15 stimulations by condition whereas the black line represents the average EMG signal by condition. The stimulation is set at Time 0 (the vertical line). Arrows, respectively, show the short- and medium-latency responses.

**FIGURE 3:**
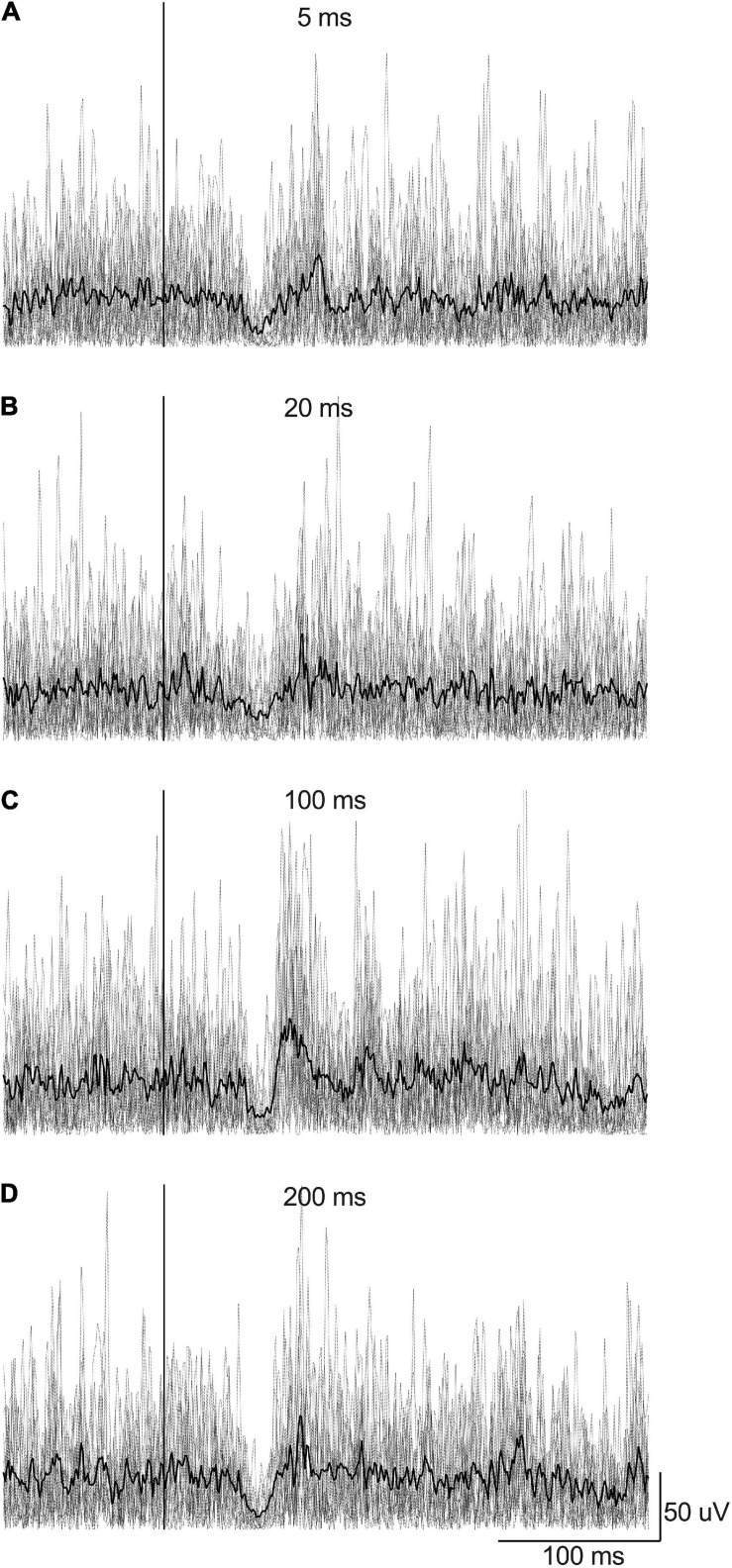
Example of rectified EMG signals recorded from the right LES muscles from a participant according to EVS duration (EVS intensity was 4 mA with right head rotation): **(A)** 5 ms, **(B)** 20 ms, **(C)** 100 ms, and **(D)** 200 ms. Gray lines represent the EMG traces recorded for each 15 stimulations by condition whereas the black line represents the average EMG signal by condition. The stimulation is set at Time 0 (the vertical line).

The mean age of participants (3 males, 7 females) was 24.3 (3.4) years. LES motor responses in one representative participant are presented in Figure 2 according to current intensity, and in Figure 3 according to EVS duration. The short- and medium-latency LES motor responses are obvious, especially at higher EVS current intensity. Table 1 shows the amplitudes, occurrences and latencies of the short- and medium-latency motor responses. Note that the short-latency response was inhibitory and the medium-latency response was excitatory regardless of the direction of the head rotation for all participants.

**TABLE 1:** Mean amplitudes, occurrences and latencies of the short- and medium LES motor responses by condition [mean (SD)].

**Condition**		**Amplitude (% MVC)**		**Occurrence (%)**		**Latency (ms)**	
**mA**	**ms**	**(Short)**	**(Medium)**	**(Short)**	**(Medium)**	**(Short)**	**(Medium)**
**Right rotation (*n* = 10)**							
2	5	−1.32 (1.84)	2.75 (2.73)	10	40	28.3 (n.a.)	80.1 (22.0)
	20	−1.63 (2.03)	3.28 (2.41)	30	40	46.1 (2.6)	69.0 (12.6)
	100	−1.91 (2.33)	2.17 (2.15)	30	40	57.2 (11.8)	83.7 (9.3)
	200	−1.84 (3.05)	1.87 (3.13)	30	20	49.0 (4.7)	68.9 (14.4)
3	5	−1.94 (1.94)	2.83 (3.56)	50	60	48.9 (4.9)	69.4 (7.1)
	20	−2.22 (3.34)	4.73 (4.28)	50	80	45.0 (10.8)	67.6 (7.6)
	100	−2.70 (2.69)	3.71 (3.43)	40	30	50.9 (3.5)	73.4 (5.2)
	200	−2.95 (2.34)	3.96 (3.60)	60	60	44.3 (13.7)	69.5 (5.4)
4	5	−4.28 (1.87)	3.04 (5.12)	70	60	47.6 (6.9)	75.2 (16.5)
	20	−2.13 (3.08)	4.42 (2.84)	60	70	46.4 (10.5)	67.4 (10.0)
	100	−3.31 (2.53)	3.74 (2.84)	70	60	50.7 (10.0)	77.7 (7.5)
	200	−3.56 (2.74)	4.33 (3.16)	70	70	47.9 (3.2)	70.4 (5.0)
5	5	−2.85 (2.47)	3.37 (4.53)	60	60	44.1 (5.9)	69.3 (14.5)
	20	−3.60 (2.78)	5.28 (3.91)	80	70	43.1 (7.0)	72.1 (4.3)
	100	−2.57 (4.10)	5.33 (2.79)	70	80	42.8 (10.8)	70.4 (12.5)
	200	−3.50 (2.98)	5.15 (3.09)	70	80	45.1 (4.2)	70.5 (4.7)
**Left rotation (*n* = 10)**							
2	5	−0.53 (1.69)	1.30 (3.29)	10	20	58.0 (n.a.)	86.3 (5.2)
	20	−0.18 (1.83)	1.87 (3.46)	0	10	n.a.	67.0 (n.a.)
	100	−3.15 (1.96)	1.50 (4.17)	60	20	39.7 (11.7)	72.3 (5.8)
	200	−0.31 (2.95)	1.82 (3.38)	10	40	48.6 (n.a.)	61.0 (12.5)
3	5	−1.09 (2.59)	2.43 (4.12)	20	40	44.7 (13.3)	70.7 (10.7)
	20	−2.80 (1.97)	3.76 (3.94)	50	50	51.2 (2.7)	73.4 (8.4)
	100	−2.53 (2.55)	2.03 (4.75)	40	30	55.0 (21.3)	68.2 (4.3)
	200	−1.62 (2.37)	1.56 (2.16)	10	30	52.9 (n.a.)	76.7 (19.7)
4	5	−3.05 (2.59)	3.31 (4.31)	50	50	42.3 (4.1)	72.3 (9.5)
	20	−2.24 (2.77)	4.41 (4.27)	50	70	45.1 (5.4)	76.0 (13.1)
	100	−2.09 (2.82)	3.27 (5.92)	40	40	41.1 (9.4)	68.9 (7.9)
	200	−1.88 (3.30)	2.86 (4.03)	40	50	40.3 (8.4)	61.3 (8.6)
5	5	−2.87 (3.03)	2.78 (4.30)	50	50	44.1 (3.4)	67.8 (4.7)
	20	−3.60 (3.09)	4.75 (4.30)	60	70	46.2 (3.0)	78.6 (13.1)
	100	−3.70 (3.18)	4.92 (2.97)	70	80	41.1 (5.8)	73.2 (10.2)
	200	−2.82 (2.65)	2.09 (4.76)	50	60	45.2 (2.8)	72.2 (6.5)
**No rotation (*n* = 9)**							
2	5	−0.40 (1.16)	1.42 (3.22)	0	11	n.a.	65.0 (n.a.)
	20	−1.77 (1.92)	0.99 (3.06)	11	11	55.3 (n.a.)	68.9 (n.a.)
	100	−1.46 (2.56)	2.97 (3.40)	33	44	42.9 (21.8)	74.3 (14.5)
	200	−0.64 (1.36)	2.02 (2.12)	0	22	n.a.	84.4 (13.6)
3	5	−1.93 (2.13)	1.40 (3.63)	33	22	41.3 (10.5)	67.8 (4.5)
	20	−1.93 (2.98)	3.55 (5.20)	33	44	51.1 (3.6)	73.1 (6.2)
	100	−2.01 (1.76)	3.24 (3.29)	22	44	42.5 (0.1)	79.0 (23.0)
	200	−1.33 (2.48)	4.12 (3.08)	33	67	50.6 (5.8)	86.0 (19.9)
4	5	−1.13 (3.05)	2.37 (3.21)	44	44	36.6 (12.0)	70.2 (7.5)
	20	−1.86 (2.82)	4.02 (2.81)	44	67	41.4 (5.4)	71.8 (20.1)
	100	−2.55 (3.05)	4.99 (3.32)	67	78	51.2 (7.1)	75.3 (13.5)
	200	−0.97 (4.38)	4.73 (1.95)	44	78	41.8 (5.0)	74.3 (10.1)
5	5	−1.67 (2.51)	3.68 (3.24)	44	56	44.0 (4.2)	68.5 (4.0)
	20	−3.02 (2.84)	5.58 (3.64)	78	78	43.0 (3.2)	70.2 (5.8)
	100	−3.27 (3.58)	5.14 (2.33)	44	78	40.0 (3.3)	79.7 (13.5)
	200	−2.96 (2.99)	5.67 (3.75)	44	78	46.7 (2.7)	73.9 (7.9)

### Effect of Parameters on the Amplitude, Occurrence, and Latency of the Short-Latency Motor Response

**FIGURE 4:**
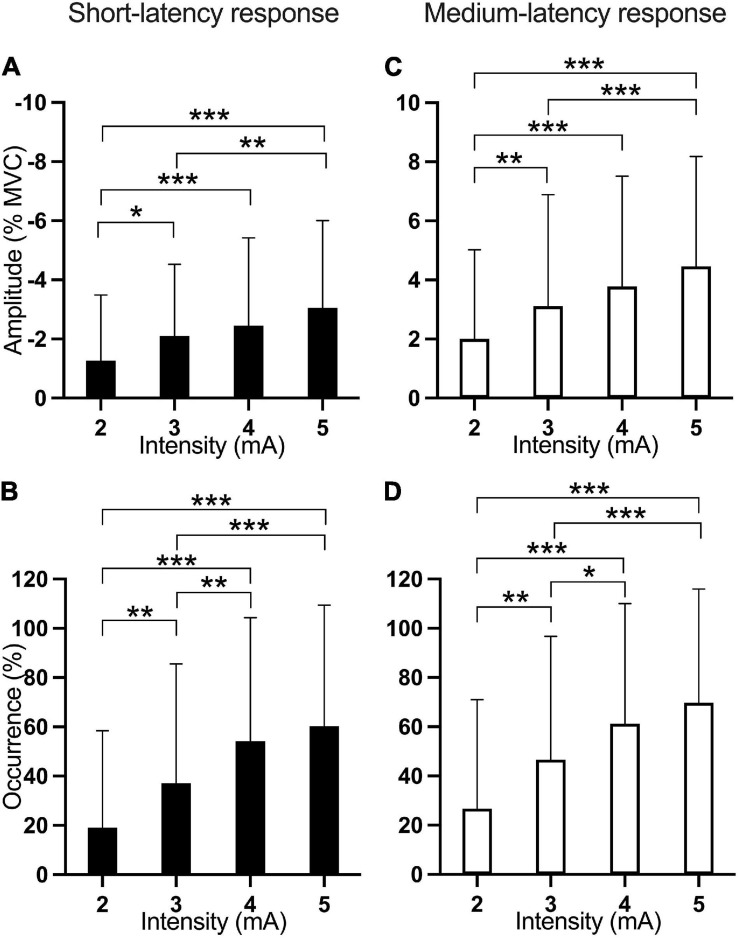
Mean amplitude and occurrence of the short- and medium-latency responses according to current intensity (rotation and stimulus duration pooled within each EVS intensity). The first column of figures **(A,B**—black) is associated with the short-latency response while the second one **(C,D**—white) is associated with the medium-latency response. The first line of figures represents the amplitude **(A,C)** of the response while the second one represents the occurrence **(B,D)**. *p < 0.05; **p < 0.01; ***p < 0.001.

The amplitude of the short-latency response showed a main effect of Current intensity [*F*_(3, 406.9)_ = 13.13; *p* < 0.001]. Pairwise comparisons show that EVS current intensities of 3 mA (*p* = 0.041), 4 mA (*p* < 0.001), and 5 mA (*p* < 0.001) produced a larger inhibition than 2 mA while current intensity of 5 mA inhibited more LES muscle activation than 3 mA (*p* = 0.004; Figure 4A).

**FIGURE 5:**
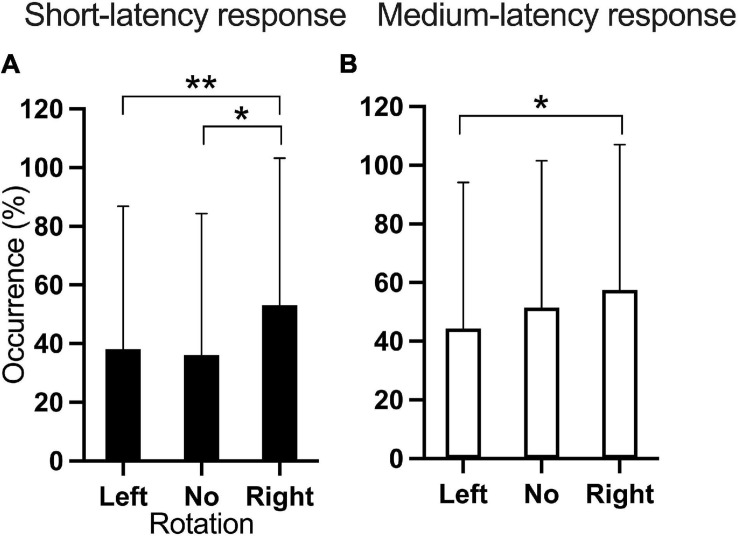
Occurrence of the **(A)** short- and **(B)** medium-latency responses according to head rotation. *p < 0.05; **p < 0.01.

The occurrence of the short-latency response showed main effects of Rotation [*F*_(2, 407.9)_ = 6.35; *p* = 0.002] and Current intensity [*F*_(3, 406.9)_ = 24.88; *p* < 0.001]. Pairwise comparisons were significant for the four EVS current intensities, except for the comparison between 4 and 5 mA (*p* = 0.841), indicating that higher EVS current intensities produced more frequently the short-latency response (Figure 4B). Also, pairwise comparisons showed that right head rotation induced more frequently the short-latency response compared to left (*p* = 0.003) and neutral (*p* = 0.02) head rotation (Figure 5A).

For all conditions combined, the mean latency of the short-latency response was 45.5 (5.3) ms. No obvious visual changes were present based on head rotation, EVS current intensity or EVS duration for the latency of the short-latency response.

### Effect of Parameters on the Amplitude, Occurrence, and Latency of the Medium-Latency Motor Response

**FIGURE 6:**
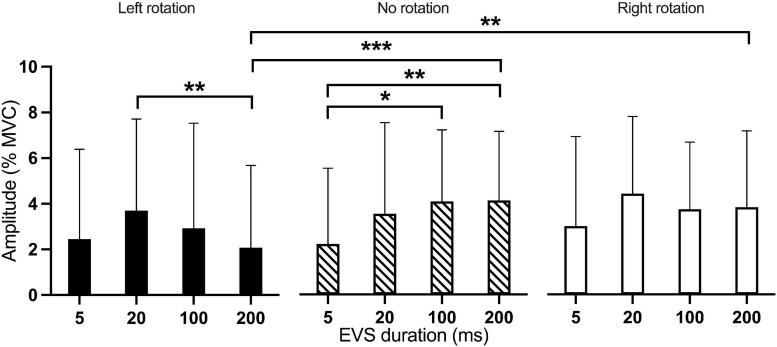
Mean amplitude of the medium-latency response according to the interaction between EVS duration and head rotation. Results of the medium-latency response are represented as amplitude against EVS durations for the three head rotations. Black bar: left rotation; hatched bar: no rotation; White bar: right rotation. *p < 0.05; **p < 0.01; ***p < 0.001.

The amplitude of the medium-latency response showed a main effect of Current intensity [*F*_(3, 407.0)_ = 20.63; *p* < 0.001] and of Rotation by EVS duration interaction [*F*_(6, 407.0)_ = 2.56; *p* = 0.019]. Current intensities of 3 mA (*p* = 0.002), 4 mA (*p* < 0.001), and 5 mA (*p* < 0.001) all produced higher responses amplitude than 2 mA. Also, 5 mA produced a higher response amplitude than 3 mA (*p* < 0.001; Figure 4C). The Rotation x EVS duration interaction showed differences between Rotations only at 200 ms; the medium-latency response was larger in right (*p* = 0.001) and neutral (*p* < 0.001) than left head rotation (Figure 6). Also, the effect of EVS duration was different depending on head rotation. With the head in neutral position, 5-ms EVS produced a smaller response amplitude compared to 200 ms (*p* = 0.008) and 100 ms (*p* = 0.011). In left head rotation, 20-ms EVS produced a larger medium-latency response compared to 200 ms (*p* = 0.006; Figure 6). With the head rotated to the right, no difference was present between EVS duration.

Rotation [*F*_(2, 408.2)_ = 3.953; *p* = 0.02] and Current intensity [*F*_(3, 407.0)_ = 23.893; *p* < 0.001] significantly affected the occurrence of the medium-latency response. Pairwise comparisons showed that Right head rotation produced a higher occurrence of the response than Left head rotation (*p* = 0.016, Figure 5B). Except for the comparison between 4 and 5 mA (*p* = 0.542), larger EVS intensities produced more frequent responses (Figure 4D).

All conditions combined, the mean latency of the medium-latency response was 73.2 (5.1) ms. No obvious changes were present based on head rotation, EVS current intensity or EVS duration for the latency of the medium-latency response.

### Effect of Vision

Vision showed no significant main effect or interaction on the amplitude [*F*_(1, 27)_ = 1.10; *p* = 0.305] or occurrence [*F*_(1, 27)_ = 1.90; *p* = 0.180] of the short-latency response, nor for the medium-latency response [amplitude: *F*_(1, 27)_ = 0.43; *p* = 0.516 | occurrence: *F*_(1, 27)_ = 0.45; *p* = 0.508].

## Discussion

This study explored the effect of EVS current intensity, EVS duration, head position and vision on the amplitude, occurrence and latency of the EVS-induced motor responses of right LES muscle. Based on our results, larger intensities of stimulation and right and neutral head rotations influenced the short- and medium-latency motor responses amplitude (i.e., more inhibited and more facilitated, respectively) and occurrence. Also, the EVS duration seemed to impact the medium-latency motor response depending on the head rotation. First, right and no head rotation compared to left head rotations produced larger motor response amplitude when using an EVS duration of 200 ms. Second, longer EVS duration produced larger medium-latency response amplitude only in no head rotation, whereas 20-ms EVS in left head rotation produced larger motor response amplitude than 200 ms. Visual cues did not influence the amplitude and occurrence of the short- and medium-latency motor responses.

### Influence of EVS Parameters on Motor Responses

Some studies reported increased amplitude of short- and medium-latency motor responses with increased EVS current intensity (Fitzpatrick et al., 1994; Day et al., 1997). Modulation in EVS current intensity may lead to the recruitment of different afferent fibers. EVS primarily activates irregular vestibular afferent fibers transmitted by fast-conducting axons rather than regular vestibular afferent fibers transmitted by slow-conducting axons (Goldberg et al., 1984; Dlugaiczyk et al., 2019). Increased EVS current intensity recruits a more significant proportion of regular vestibular afferent fibers in monkeys (Goldberg et al., 1984). In humans, the recruitment of regular vestibular afferent fibers with the increase in EVS intensity could explain the higher occurrence and amplitude of short- and medium-latency motor responses as observed in animal studies.

EVS duration can also influence the motor responses. Although EVS studies of back muscles used longer stimulus duration [e.g., 175 ms (Guillaud et al., 2020), 400 ms (Ali et al., 2003)], using stimulus duration < 100 ms also result in LES motor responses. We observed that a 5-ms EVS duration induced a medium-latency motor response of smaller amplitude than for 20, 100, and 200 ms only in no head rotation condition. With the head rotated toward the right, the medium-latency motor response amplitude did not differ between EVS durations. In contrast, with the head toward the left, the medium-latency motor response amplitude was larger in 20 ms compared to other EVS durations. There was no effect of EVS duration on the short-latency motor response amplitude. Explaining the differences between the short- and medium-latency motor responses in different head positions remains challenging.

Overall, our results suggest that 5-ms EVS duration may induce motor responses, even though in few conditions their amplitude was smaller. EVS duration of 20 ms compared to 100- and 200-ms induced equal or larger motor responses, depending on the condition. Other studies reported that brief EVS duration might evoke responses. For example, EVS of 2-ms duration (5 mA) can evoke motor responses in the sternocleidomastoid (Watson and Colebatch, 1998) and masseter (Deriu et al., 2003) muscles, although they were only associated with movement of the head relative to the trunk rather than whole-body sway.

### Influence of the Visual System

The EVS-induced motor response amplitude is highly context-dependent (Day et al., 1997; Fitzpatrick and Day, 2004; Gandevia et al., 2012; Maaswinkel et al., 2014; Ertl and Boegle, 2019). Biasing visual or proprioceptive input is frequently used to test their contribution to postural control. For example, if vision contributes to the motor responses, the absence of vision should increase its amplitude. In presence of visual input, the central nervous system could process the vestibular information induced by EVS as unphysiological or aberrant considering the mismatch between inputs coming from these sensory systems and reduce the gain on vestibular information. Vestibular-visual interaction occurs in standing, as authors reported much smaller EVS-induced motor responses with eyes open compared to closed (Britton et al., 1993; Fitzpatrick and Day, 2004). In contrast, our results do not support a significant contribution of vision on the amplitude and occurrence of the short- and medium latency motor responses in sitting. Increased gain of the somatosensory information while sitting could explain the small contribution of vision. In line, Fitzpatrick et al. (1994) concluded that visual input had a minor effect on EVS-induced responses when more somatosensory cues were available. In sitting, compared to upright standing, the large contact of the thighs and pelvis with the seat (i.e., involving more tactile receptors) may have provided large somatosensory input making postural control easier, even in the absence of vision. Future studies should test this hypothesis.

### Patterns of the Short- and Medium-Latency Motor Responses

In an upright standing position, short- and medium-latency responses in *soleus* muscles (Britton et al., 1993; Fitzpatrick et al., 1994) are opposite (i.e., inhibitory or excitatory). The direction of the response depends on the rotation of the head relative to the position of the anode/cathode. Britton et al. (1993) reported an inhibitory short-latency response followed by an excitatory medium-latency response in the *soleus* when the experimental conditions produced a backward sway (left head rotation and anode on the left). Opposite head rotation or the same rotation with the anode on the opposite mastoid process reversed the direction of the responses. Such a reversal did not occur in the current study: the short-latency response was always inhibitory and the medium-latency response, excitatory, regardless of the head rotation. A recent study from Guillaud et al. (2020) also reported no reversal of the short- and medium-latency LES motor responses when changing the polarity of the electrodes while standing with right head rotation. In another study, authors reported a reversed direction of LES motor responses by depicting EMG data from a single participant in frontal and sagittal planes of motion. However, it remains unclear whether the reversal was consistent across participants since no mean data or descriptive statistics were provided (Ali et al., 2003). This discrepancy could be explained by the sitting posture that was different between studies and the position of the electrodes that may capture the EMG signal from different back muscles having different biomechanical roles in lumbar spine control (i.e., prime mover vs. intervertebral control).

Although we did not measure body sway in sitting, Day et al. (1997) measured it and observed body sways in opposite directions when reversing EVS polarities, even though the amplitude was smaller compared to standing. Reversing EVS polarities (i.e., switching the anode and the cathode position) while keeping the same head rotation reverses the direction of body sway in the same manner than keeping the same EVS polarity but rotating the head rotation in the opposite direction. We suggest that the lack of reversal (i.e., the short-latency motor response was inhibitory and the medium-latency motor response was excitatory regardless of the direction of the head rotation) may reflect the biomechanical role of the LES muscle during body sway, i.e., that the central nervous system did not use the LES muscles as agonists of the body sway but rather as *“controllers”* of the lumbar spine posture. This hypothesis needs to be tested in future studies while recording multiple trunk/hip muscles on both sides of the body.

Although reversal was absent, head rotation modulated the amplitude of both short- and medium-latency motor responses. Indeed, the left head rotation produced smaller responses than right and no head rotations. A reduction in motor responses amplitude was observed during a condition corresponding to a forward body sway (e.g., left head rotation and anode placed to the right) compared to backward sway (e.g., right head rotation and anode placed to the right) (Ali et al., 2003; Guillaud et al., 2020). However, it remains difficult to compare the motor strategy in no head rotation with the available literature since we did not measure motor responses for LES on both sides.

### Latencies and Pathways Underlying EVS Induced Motor Responses

In paraspinal muscles, the medium-latency response ranged between 47 and 110 ms (Ardic et al., 2000; Ali et al., 2003; Guillaud et al., 2020). We observed a similar range of the medium-latency motor response (range: 61.0–86.3 ms). No previous study reported the latency of the short-latency motor response. However, since measuring latencies for each condition was not possible, we did not perform statistical analysis for this parameter. Although studies suggested that an increase in EVS current reduced the motor response onset (Iles and Pisini, 1992; Rosengren and Colebatch, 2002; Ali et al., 2003), we did not observe such a trend.

EVS depolarizes the eighth cranial nerve axons, and the action potentials travel to the muscles through the vestibulospinal tract (for review Forbes et al., 2014). The latencies observed for EVS-induced motor responses are 45.5 (5.3) ms for the short-latency response and 73.2 (5.1) ms for the medium-latency response. These relatively long latencies do not correspond to a monosynaptic fast-conducting pathway even when considering the latency between the vestibular organ and the vestibular nuclei. For example, the latency of the LES motor evoked potential (MEP) by transcranial magnetic stimulation of the primary motor cortex is between 14 and 18 ms (Tsao et al., 2011a; Jean-Charles et al., 2017). This latency is considered to represent a monosynaptic connection between the corticospinal cell and the α-motoneuron at the spinal level (Ferbert et al., 1992). Some authors argue that a long duration of central processing (e.g., by larger networks of interneurons at the vestibular nuclei and spinal cord levels) may cause these longer latencies (Forbes et al., 2014). For example, there is evidence in animal models that the vestibular pathways form direct (excitatory) or indirect (excitatory or inhibitory) connections with α-motoneurons (Lund and Pompeiano, 1968; Wilson and Yoshida, 1969; Grillner et al., 1970; Shinoda et al., 1986; Davies and Edgley, 1994). In other hand some could argue that the long latency of the EVS responses could be related to a late depolarization of the vestibular afferents due to long stimulus durations that are often used in EVS studies. However, the similarity of latencies (i) across EVS stimulus durations and (ii) between studies reinforce the validity of the observed EVS motor response latencies. Altogether, these results suggest that central processing of the vestibular afferents may occur at multiple levels of the central nervous system.

### Limitations

Results need to be interpreted considering different methodological aspects. Even though attenuated by the application of anesthetic cream, tactile sensation induced by EVS can affect the responses (Ertl and Boegle, 2019). Moreover, the small number of participants combined with the large number of analyses carried out may have increased the likelihood of type II and I errors. EMG activity was only recorded in the right LES muscle, which prevented to confirm the absence of motor response reversal in the no head rotation condition (i.e., in the frontal plane of motion). Since the center of pressure or the trunk kinematics was not recorded, the analysis of the relationship between body sway and head rotation is based on the results of another study (Day et al., 1997). The number of stimulations per condition may be considered quite small when comparing to other studies (e.g., 160 stimulations; Ali et al., 2003). However, discernible motor responses without the need to integrate the EMG signal were observed in all participants. Also, the use of 15 conditions allowed to attain the study objectives, i.e., to identify optimal parameters. However, it is possible that more stimulations would have allowed to observe motor responses of smaller amplitude in some participants. Considering that not all conditions were repeated with eyes open, it is possible that vision could alter LES motor responses using untested EVS parameters and conditions. It is not possible to completely exclude the presence of an off-response for the 5 ms and the 20 ms-conditions, even though the results suggest a weak effect, if any, based on similar pattern of response and latency between EVS durations. We did not standardize head rotation between participants, considering the variability in individual cervical spine range of motion. Different amplitudes of head rotation between participants may have influenced the amplitude of the EVS-induced motor responses. However, we standardized head rotation (left vs. right) within-participant using the chin-acromion distance, which is rarely done in EVS studies.

## Conclusion

This study explored the effect of head rotation, intensity, and duration of the EVS current, and vision on the EVS-induced motor responses in LES muscles. The EVS current intensity and EVS duration influenced the short- and medium-latency motor responses amplitude and occurrence. No reversal of the short- and medium-latency motor responses occurred in opposite head rotation (right vs. left). We suggest that this reflects the biomechanical role of the LES muscles to fine-tune the position of the lumbar lordosis during induced body sway. Finally, the presence of vision did not modulate the motor response amplitude and occurrence, suggesting a minimal contribution of vision to vestibulomotor control in sitting position.

## Data Availability Statement

The raw data supporting the conclusions of this article will be made available by the authors, without undue reservation.

## Ethics Statement

The studies involving human participants were reviewed and approved by the Ethics Research Committee of the Centre Intégré Universitaire de Santé et de Services Sociaux de la Capitale-Nationale. The patients/participants provided their written informed consent to participate in this study.

## Author Contributions

HM-A, MS, and AD conceived the idea for the manuscript. J-PC elaborated a microcontroller, necessary to collect data, and provided essential technical assistance. MD and AD processed to the recruitment of participants and the collection of data. AD and HM-A performed the data analysis. All authors were involved in drafting the article and or revising it critically for important intellectual content.

## Conflict of Interest

The authors declare that the research was conducted in the absence of any commercial or financial relationships that could be construed as a potential conflict of interest.

## Abbreviations

EMG: Electromyography
EVS: Electrical vestibular stimulation
LES: Lumbar *erector spinae* muscles
MVC: Maximal voluntary contraction
rms: Root mean square.
